# Shear Strength Characteristics of Recycled Concrete Aggregate and Recycled Tire Waste Mixtures from Monotonic Triaxial Tests

**DOI:** 10.3390/ma14237400

**Published:** 2021-12-02

**Authors:** Katarzyna Gabryś, Algirdas Radzevičius, Alojzy Szymański, Raimondas Šadzevičius

**Affiliations:** 1 Water Centre WULS, Warsaw University of Life Sciences—WULS, 6 Ciszewskiego Street, 02-787 Warsaw, Poland; 2 Department of Water Engineering, Faculty of Engineering, Vytautas Magnus University, Universiteto Street 10, Akademija, LT-53361 Kaunas, Lithuania; algirdas.radzevicius@vdu.lt (A.R.); raimondas.sadzevicius@vdu.lt (R.Š.); 3 Department of Geotechnics, Warsaw University of Life Sciences—WULS, 159 Nowoursynowska Street, 02-787 Warsaw, Poland; alojzy_szymanski@sggw.edu.pl

**Keywords:** recycling, environment, waste, anthropogenic soil, laboratory tests, mechanical properties

## Abstract

Recycled concrete aggregate (RCA) is a promising substitute for natural aggregates and the reuse of this material can benefit construction projects both economically and environmentally. RCA has received great attention in recent years in the form of aggregate as well as a geotechnical material of sand size. Next to RCA, another recycled material, which reduces the waste volume and is a part of the present challenges in civil engineering, is tire waste. Despite the good engineering properties of recycled tire waste (RTW), its use is still limited, even after almost 30 years since they were first introduced. To broaden the applicability of reused concrete and rubber, a further understanding of their properties and engineering behavior is required. For this reason, the main subject of this paper is composite materials that consist of anthropogenic soil recycled concrete aggregate (RCA) and crushed pieces of recycled tire waste (RTW). In this study, a series of isotropic consolidated drained triaxial tests were undertaken to characterize the shear strength of eight mixtures of variable grain-size distribution, rubber inclusion (RC), and fine fraction (FF) content. The results show that the introduction of rubber waste leads to changes in the strength parameters of the tested mixtures. Improvements in RCA shear strength were observed, the largest for the mixture M7 with 10% of recycled tire waste. Similarly, the effect of fine fraction content on the angle of internal friction and cohesion was found. Dilation characteristics were observed in all analyzed composites. Based on the results of all tests performed, including physical, geometric, chemical, and mechanical properties of the created composites, it can be stated that the samples would meet local road authority requirements for sub-base applications.

## 1. Introduction

The modern civil engineering industry requires the continuous development of new technologies and materials. Because of the already existing deficiencies of aggregates and natural soil in various regions of Poland, as well as unfavorable forecasts for the market, the possibility of replacing natural aggregate with recycled materials becomes a good alternative.

Recycled concrete is a material that can successfully merge economic and environmental interests. The current prevalence of recycled concrete and the scale of demolition together with modernization work in the construction industry indicate that this material will long be a potential material for earth structures [1]. There are researchers that emphasize the fairly large aggregates recycling acquisition costs, the lack of homogeneity, and the need to introduce additional processes that significantly affect the economics of commodity production [2]. On the other hand, the use of recycled materials is undoubtedly a good way to go, because of the aforementioned environmental protection or the high energy expenditure of extracting natural aggregates, and the high price of transporting them.

A huge problem in the world, as well as in Poland, is the storage and management of rubber waste [3]. Currently, there are two groups of rubber waste, in which the first group includes vehicle tires (about 70%), whereas the second group is industrial waste and conveyor belts (around 30%) [4]. Worn tires are reconditioned, recycled, or burned in cement plants. At present, it is estimated that there are over 29 million tons of used tires in the world, 23% of which are recycled.

Shredded used tires are now being used in landfill engineering as subgrade reinforcement for constructing roads over soft soil, as well as aggregate in leach beds for septic systems, and as a substitute for leachate collection stone in landfills [5]. Crumbed or shredded used tires are applied as an energy-producing material, an admixture in bituminous concrete, and low-grade rubber products, e.g., truck-bed liners, doormats, and cushioning forms [6]. Whole rubber tires can be also used as reinforcement in the construction of retaining walls and slopes [7]. Another possible practical application of shredded tires alone or mixed with soil is as a lightweight material for embankment fill. In general, the use of shredded rubber waste depends on the degree of shredding. In the European Standard EN-14243, materials obtained from the recycling of tires are classified as shown in Table 1.

It is worth emphasizing that the economic aspects are at the moment a barrier to the wide introduction of the utilization of rubber waste from worn vehicle tires to the Polish market. However, in connection with the introduction of a ban on burning waste, the situation may radically change shortly.

In Table 2, a comparison between the basic parameters of recycled tire waste in the form of rubber aggregate and mineral soil packages is presented. To reduce the size of landfills and thus reduce the high fire risk, tires began to be packaged in packages reducing the volume of stored materials by more than four times. An attractive construction form of recycled material in the form of rubber packages (gabions) was obtained to employ in many various constructions [9].

This study examined only the shear strength characteristics of recycled concrete aggregate (RCA) with recycled tire waste (RTW). Shear strength is a fundamental mechanical property that determines, e.g., the stability of embankment structures. The higher the value of the friction angle, the higher the soil shear resistance, the lower the earth pressure, the higher the stability. Moreover, recent geotechnical investigations on soil-rubber mixtures focus generally on the effects of rubber on the mechanical behavior of natural clean sands, silts, low-plasticity clays, gravels, and anthropogenic fly ashes. Several studies have been conducted as well to evaluate the effect of crumb rubber on the properties of cohesive, frequently expansive, soils. Unfortunately, very few tests have been done to examine the properties of recycled concrete aggregate and rubber waste composites.

Experiments on sand mixtures with rubber chips and rubber shreds by the international community date from the 1990s, when Edil and Bossher [10] and Wu et al. [11] examined the strength of those materials in triaxial apparatus. Mechanical properties of crushed tires were also later evaluated by Yang et al. [12] with isotropic and confined compression, using direct shear and triaxial tests. Various attempts to obtain optimum tire content with the maximum shear strength of the mixture resulted in nearly 35% by weight [13].

The inclusion of shredded tires to clay soils showed a significant decline of friction angle by approximately 22%, according to consolidated-drained triaxial tests [14]. In the case of rubber addition to noncohesive soils, like sand, a decrease of friction angles up to even 30% was observed in the direct shear apparatus tests [15]. The change in friction angle was accompanied by changes in cohesion. Bałachowski and Gotteland [16] detected an increase in the apparent cohesion of the sand–rubber mixtures, by approximately 50% of rubber waste content. Similar observations had Kowalska and Chmielewski [15] but at 30% of rubber content. When these values were exceeded, cohesion, after an initial increase, began to show a decreasing tendency. Thus, to achieve the highest strength of the mixture, the proper proportion between the values of strength parameters and the content of the rubber additive is always searched for.

When it comes to mixing RCA with RTW, in 2015, our group [17] began to study the physical and mechanical properties of geocomposites composed of recycled concrete aggregate and crushed car tires at different concentrations, namely 0%, 0.5%, 1% by weight. However, these studies were performed under cyclic loading conditions (cyclic CBR), and the results indicated an increase in the elasticity modulus (E and M_r_) and a decrease in plastic deformations with the rubber content in the analyzed material. The subsequent studies concerned further mixtures of RCA with rubber waste [18], but this time RCA was improved with recycled rubber powder in the amount of 0% and 15%. The results showed that addition of tires significantly decreased the value of shear wave velocity—and consequently the values of the shear modulus—of the modified RCA.

To the authors’ knowledge, very little attention has been paid so far to the strength and deformation characteristics of modified RCA by shredded rubber tires. Therefore, the primary purpose of the presented paper is to evaluate the friction angle (φ) and cohesion (c) of the recycled concrete aggregate and granulated rubber geocomposite with various rubber contents/sizes and under different normal stresses. This study focuses on the experimental investigation of strength characteristics of eight different mixtures. Isotropic consolidated drained (CID) tests were performed on samples with the different mixing ratios of RCA and shredded rubber tires.

## 2. Materials and Methods

### 2.1. Description of Materials

**Figure 1:**
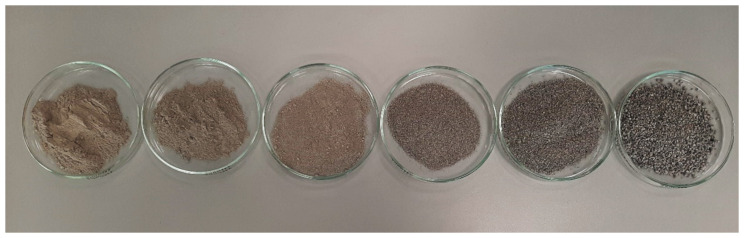
Grain fractions of concrete aggregate, from the smallest from the left: <0.063 mm; 0.063–0.125 mm; 0.125–0.25 mm; 0.25–0.50 mm; 0.5–1.0 mm; 1.0–2.0 mm.

In this study, demolished concrete from building demolition sites and industry-produced scrap tires from Warsaw, Poland were used. Concrete aggregates were an element of concrete floors, especially concrete curbs from the demolition of roads. This material is about 20 years old. The strength class of concrete from which the aggregates were formed was estimated between C16/20 and C30/35. The aggregates were 99% broken cement concrete by weight and 1% glass and brick (Σ(Rb, Rg, X) ≤ 1% m/m), following the Polish Committee for Standardization [19]. They did not contain asphalt or any tar elements. The basic properties of the original concrete were determined for the authors’ earlier study and can be found in their other publications [20]. Concrete aggregates required preliminary research on saturation to prevent the movement of water necessary for hydration [20]. The material did not show any binding properties. Recycled concrete aggregates (RCA) needed for the specimens are shown in Figure 1.

After obtaining sufficient quantities of the selected fractions (Figure 1), four different mixtures of RCA were created with appropriate compositions and the following percentages of the fine fraction (FF), i.e., <0.063 mm:

➢ M1_RCA_0FF,
➢ M2_RCA_10FF,
➢ M3_RCA_20FF,
➢ M4_RCA_30FF.

**Figure 2:**
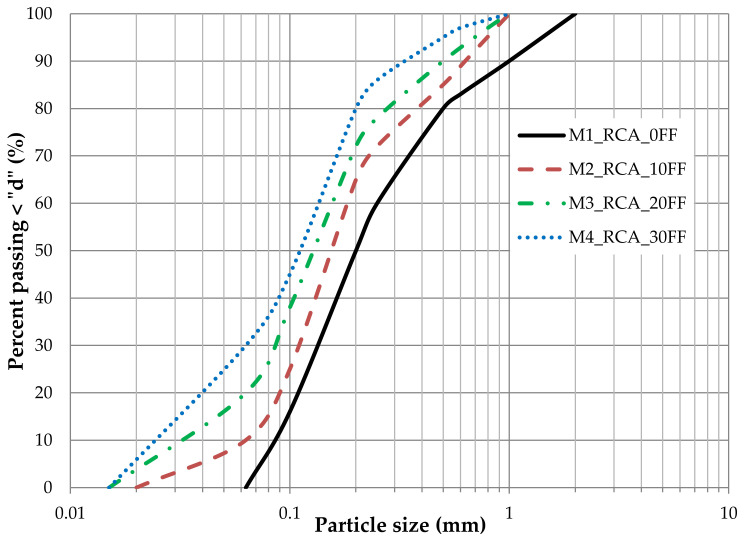
The particle size distribution curves of M1, M2, M3, and M4 used for this study.

The particle size distribution curves of all four mixtures are shown in Figure 2.

The soil was classified as sand (Sa) for mixtures M1_RCA_0FF and M2_RCA_10FF and sand with silt (siSa) for mixtures M3_RCA_20FF and M4_RCA_30FF, following PN-EN ISO 14688-2:2006 [21]. The main properties of these mixtures of RCA are given in Table 3. The specific gravity for all used materials was found to be 2.62.

When evaluating the tested four RCA mixtures for coefficients of the grain size distribution (C_u_, C_c_, d_10_), the two first (M1 and M2) can be classified as poorly graded sands. Mixtures M3 and M4 are classified as well-graded sands with silt. All tested mixtures are of uniform grain size.

In addition, selected physical and geometric tests were performed on the properties of the concrete aggregates used to create all the mixtures analyzed in this study. The results of these tests are presented in Table 4.

The laboratory physical properties evaluation also included compaction tests to characterize the optimum moisture content (OMC) and the maximum dry density (ρ_d max_) of RCA by the Proctor method. The details regarding this method have been presented in [22]. The following results were obtained: the average value of OMC is 7.4%, the uncertainty of measurement is 6.3%, and the average value of ρ_d max_ is equal to 1.69. The uncertainty of measurement is 2.5%.

The chemical compositions of RCA blends are listed in Table 5. In addition, the analysis of concentrations of chlorides, sulfates, and heavy metals in all tested materials was carried out. The methodology of these studies has been described in [23]. In Table 6 the results from conducted leaching tests of RCA blends are presented.

Considering the physical, geometric, and chemical properties of the recycled aggregate used to create the M1–M4 mixes, the potential application of the proposed mixtures could be as a supportive road subbase.

**Figure 3:**
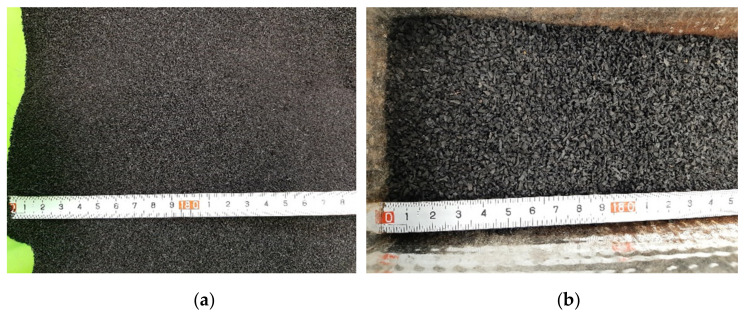
Rubber waste: (**a**) powder 0.5–1.0 mm; (**b**) granulate 1.0–2.0 mm.

Ground rubber waste from worn vehicle tires applied in the presented research was shredded and processed in the local tire manufacturing plant. Two sizes of tire waste were used: powder with the diameter size respectively 0.5–1.0 mm (Figure 3a) and granulate with dimensions 1.0–2.0 mm (Figure 3b). Both rubber additives did not contain any amounts of textile parts or steel belts. Both applied rubber wastes are not harmful to health. According to the manufacturer’s information, these products have stable composition and do not pose a threat during storage and transport. They have been positively assessed in terms of health by the national institute of hygiene.

The basic components of used waste tires are (% mass/tire): natural rubber 15%, SBR 20%, BR 10%, IIR/XIIR 5%, silica 15%, carbon black 15%, sulfur 2%, resin 2%, mineral and vegetable oils 10%, others (zinc oxide, stearic acid) 6%. The presence of heavy metals in tires is related to the technological process. Here, these are at very negligible amounts.

### 2.2. RCA–RTW Mixtures

To prepare the appropriate compositions of ingredients, the exact amount of RCA and RTW was estimated. Four mixtures were developed with varying percentages of recycled rubber content. All samples were prepared by careful hand mixing of rubber with recycled concrete aggregates, so the mass of granulate and/or powder was appropriately 20%, 15%, 10%, and 5% of the total mass. The authors limited this study to mixtures with maximum rubber content equal to 20% by weight, because it has been reported in the literature that for rubber content more than 30–35% by weight, the mixtures exhibit in general rubberlike behavior. This is mainly due to the predominant development of rubber-to-rubber interfaces and thus the overall static and dynamic responses of the mixtures are then mainly controlled by the rubber part [24].

**Figure 4:**
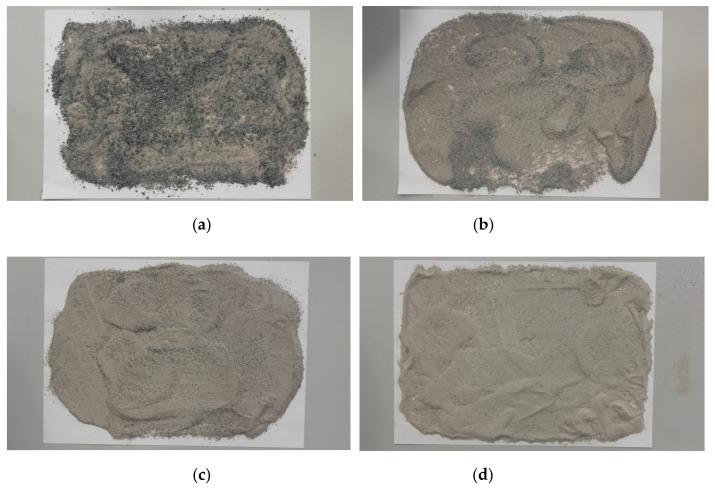
A view of RCA–RTW mixtures. (**a**) M5_RCA_20R_0FF; (**b**) M6_RCA_15R_10FF; (**c**) M7_RCA_10R_20FF; (**d**) M8_RCA_5R_30FF.

**Figure 5:**
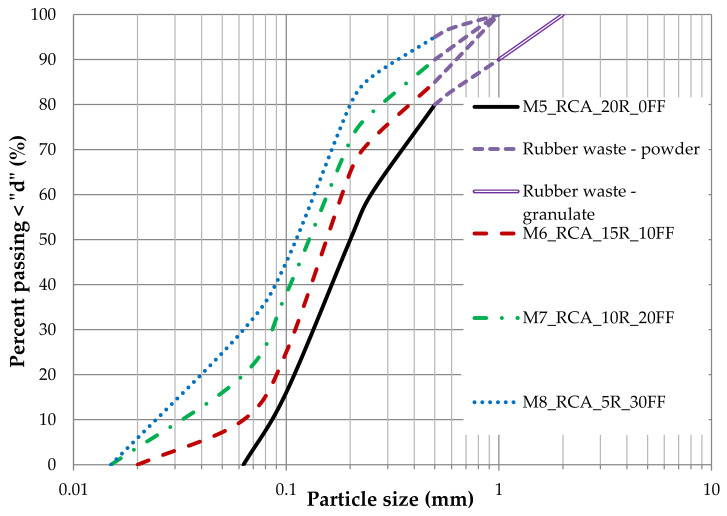
The particle size distribution curves of M5, M6, M7, and M8 used for this study.

The basis for forming new blends discussed here were the previously mentioned four RCA compositions (M1, M2, M3, and M4). The new blends, shown in Figure 4, were created in such a way that the corresponding RCA fraction, with grain sizes ranging from about 2.0 mm or 1.0 mm to 0.5 mm, was eliminated and replaced with rubber waste. The particle size distribution curves of all four RCA–RTW mixtures are shown in Figure 5. It is worth noting that as the content of the fine fraction (FF), i.e., <0.063 mm, increases, the content of waste rubber decreases. The mixes created thus have the following compositions:

➢ M5_RCA_20R_0FF (RCA with 10% addition of 0.5–1.0 mm powder, 10% addition of granulate 1.0–2.0 mm and 0% of RCA fine fraction),
➢ M6_RCA_15R_10FF (RCA with 15% addition of 0.5–1.0 mm powder and 10% of RCA fine fraction),
➢ M7_RCA_10R_20FF (RCA with 10% addition of 0.5–1.0 mm powder and 20% of RCA fine fraction),
➢ M8_RCA_5R_30FF (RCA with 5% addition of 0.5–1.0 mm powder and 30% of RCA fine fraction).

These mixes could potentially be used in the construction of road embankments. The idea would be to replace the traditional natural soil with a material derived from recycling so that it performs the same functions as the traditional soil.

### 2.3. Testing Methods

A series of isotropic consolidated drained (CID) triaxial tests were performed to evaluate the effect of the percentage of recycled tire waste on the strength characteristics of eight different specimens. The automated triaxial testing system adopted for this study is described in the following papers [18,25]. The specimens were prepared in the laboratory using the measurement of weight. The exact amount of RCA and RTW were mixed by hand thoroughly until a uniform mixture was achieved. Next, the mixtures were transferred to a triplicate mold, tightly coupled together, with a rubber membrane wound on the inside and secured with two O-rings. This mold was set on a pore stone additionally equipped with a filter paper, based on a triaxial apparatus. The mixtures were placed in the mold in layers and special care was taken that the rubber does not segregate. Each layer was compacted by a small hand tamper, to obtain specimens in a compacted state. When the specimens were completely prepared, standard initial steps of triaxial tests were performed, i.e., flushing, saturation, and consolidation. All the mixtures were tested at three different effective stresses, i.e., 90, 180, and 270 kPa (covering a range of pressures expected in many geotechnical applications [26]). At the end of consolidation, the axial load was increased at a constant rate of axial strain until an axial strain of 15% was reached or failure occurred. The shear rate of each specimen was set at 0.033 mm/min based on the oedometric tests performed and the experience of the researchers. During shear, data of axial deformation and axial load were recorded from the dial gauges and load cell, respectively.

## 3. Testing Results and Discussion

Shear strength characteristics and volumetric characteristics of the composite materials were examined concerning the percentage of rubber waste and the applied effective stress on the specimens in the CID triaxial tests. The possible influence of rubber addition on the cohesion and friction angle of RCA were analyzed considering the contents of rubber. Additionally, the influence of the fine fraction of recycled concrete aggregate on the shear properties was also studied. In this section, the results of laboratory tests are presented with a discussion highlighting the effects of the various parameters. The inclusion of all figures in this paper is cumbersome and makes the paper long. Only a limited number of them are presented here. Additionally, each composite was tested several times and the results presented are averaged.

### 3.1. Stress-Strain Behavior

**Figure 6:**
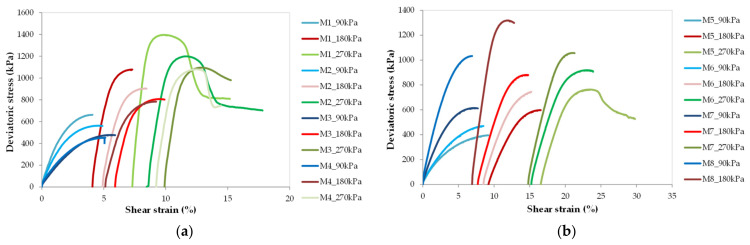
stress-strain characteristic for RCA and RCA–RTW mixtures. (**a**) RCA mixtures: M1, M2, M3, and M4; (**b**) RCA–RTW mixtures: M5, M6, M7, M8.

Typical stress-strain plots from CID triaxial test for all blends created, at the mean effective stresses of 90, 180, and 270 kPa, are shown in Figure 6a,b. Blue color indicates data for 90 kPa, red for 180 kPa, and green for 270 kPa. From Figure 6, we can observe a relatively clear peak in shear resistance for all mixed specimens, regardless of rubber contents. For the mixtures not containing tire waste (see Figure 6a), the deviatoric stress is the highest for the M1 specimen, i.e., the one without a fine fraction of RCA, whereas the smallest for M4 specimen, i.e., the mixture with 30% of the fine fraction of RCA. At the same time, the smallest values of the shear strain at failure are obtained for M1 mixtures and the highest for the M4 blend. However, analysis of Figure 6b shows that for mixtures of modified recycled concrete aggregate, it is exactly the opposite as for the composites M1–M4. The addition of rubber waste does not increase the deviatoric stress. After comparing the stress-strain characteristics from Figure 6b, we found that with decreasing rubber content and increasing fine fraction content, the shear resistance of the mixtures increases by about 20% with shear strain at failure also decreasing by about 20%. The exception is the M8 mix, which has only been tested at two mean effective stresses, namely p’ = 90 and 180 kPa. Thus, it appears that fine fraction content next to rubber content influences the shear strength of the mixtures.

On the other hand, comparing with Figure 6a,b, it can be seen that the initial slope of the stress-strain curves of RCA–RTW mixtures at 90 kPa is greater than the stress-strain curves of RCA specimens at the same confining pressure. For the other tests, at higher pressures, no such trend is observed. This can indicate that recycled concrete aggregate modified by rubber addition will have more strength but only at small strain values.

**Figure 7:**
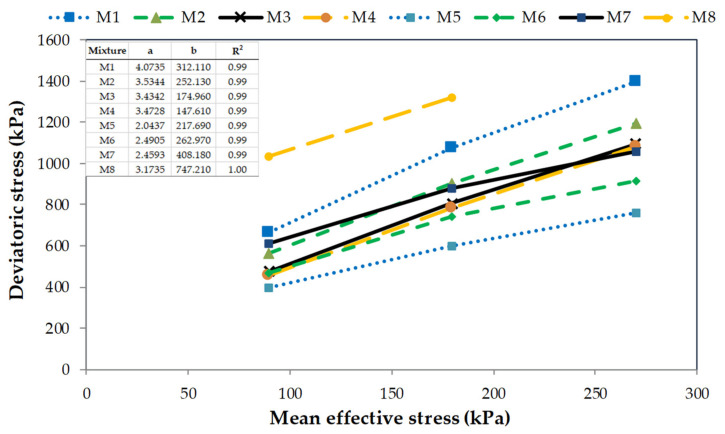
Deviatoric stress versus mean effective stress for all tested mixtures.

In Figure 7, the variations of deviatoric stress versus mean effective stress (p’) for all studied blends are presented. For all tested compositions, with increasing p’ the higher deviatoric stresses are obtained. It can be observed a linear relationship between deviatoric stress and mean effective stress. Very high values of the coefficients of determination (R^2^) show a very good adaptation of this model to the experimental data. In addition, the effect of strengthening is not observed in all cases in Figure 7. It was expected that the addition of rubber grains can significantly enhance the shear resistance of RCA, whereas only for two specimens, M7 and M8 with 10% and 5% of rubber insert respectively, this conclusion can be drawn. For the other two compositions, M5 and M6 with 20% and 15% of rubber content respectively, the maximum deviatoric stresses are, however, found for the blends made of recycled concrete aggregate alone (M1 and M2). Most likely, that is related to the fine fraction content of RCA and, on the other hand, to the method of specimen preparation. Higher content of the fine fraction, and a lower content of rubber waste, allow for a better, tighter arrangement of grains and soil particles. The fine fraction of RCA with dimensions <0.063 mm fills voids better than rubber waste of 0.5–2.0 mm. This results in better-compacted mixtures. However, it should be remembered that the content of the fine fraction is not high, the maximum is 30%. So, in the presented study, there are still materials called sand-like soil [27].

**Figure 8:**
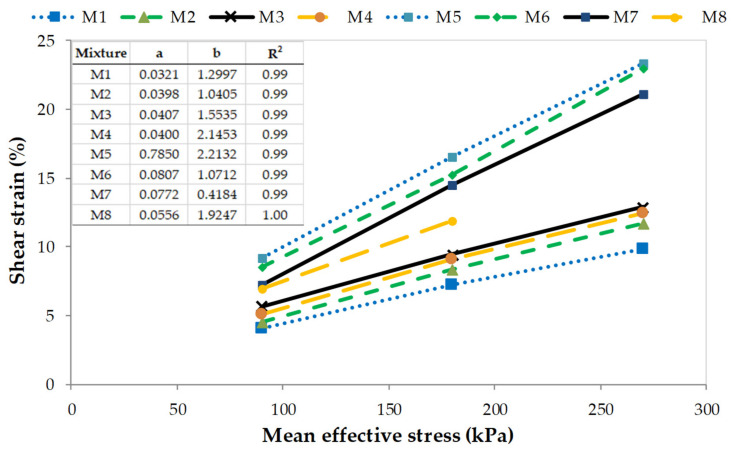
Variation of the shear strain corresponding to peak shear stress with change in mean effective stress.

A typical plot of the variation of the shear strain corresponding to peak shear stress at different confining pressure is shown in Figure 8. Due to the high deformation of the rubber waste particles, the magnitude of the shear strain at the peak deviator stress increases with the increasing proportion of rubber in the mixture. The highest values of the shear strain, regardless of the mean effective stress values, are observed for the M5 mixture, with 20% of rubber addition. As the rubber content of the specimen decreases by 5%, the strain decreases by an average of 8%. For the compositions without RTW, M1–M4, the shear strain decreases by about 50% for specimen M1 to about 25% for specimen M4.

Furthermore, it can be seen from Figure 8, that the shear strain at failure shows a linear relationship with the effective stress. In Figure 8, additionally, the summary of the linear function constants (a, b) and R^2^ is included. Analyzing the slope of the shear strain–mean effective stress curves, it can be noticed that for RCA–RTW mixtures it is similar, regardless of the rubber content, and is about five degrees. Only for sample M8, it is smaller, three degrees. RCA mixtures M1–M4, have the initial slope of the shear strain–mean effective stress curves slightly smaller, by about half in comparison with other specimens. Such small differences in slope values do not allow to unequivocally state a positive effect of the rubber additive on the shear strength of the studied mixes.

**Figure 9:**
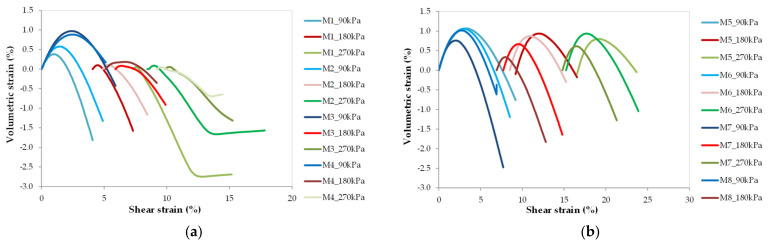
Typical plot for variation of volumetric strain with shear strain for RCA and RCA–RTW mixtures. (**a**) RCA mixtures: M1, M2, M3, and M4; (**b**) RCA–RTW mixtures: M5, M6, M7, M8.

The relationships between shear strain and volumetric strain are plotted in Figure 9. Figure 9a only provides the results of RCA specimens, whereas in Figure 9b the results of RCA–RTW mixtures are presented. The color coding corresponds to that in Figure 6. From Figure 9a, it can be seen that there is an initial expansion, i.e., dilation, and then compression, with an increase in shearing strain. The dilatancy characteristics of studied RCA blends are similar to the behavior of cohesionless material, having both negative and positive magnitudes of dilatancy [11,28]. In addition, with increasing confining pressure, the tendency of dilation decreases with increasing shear strain.

Analyzing Figure 9b, the deformation characteristics of rubberized RCA composites are very similar to each other, not depending on the mean effective stress. It is difficult to notice here a clear relationship between volumetric strains and varying rubber content, because of the very close strain values obtained. After a closer look at the values, it can be eventually concluded that volumetric strain decreases little with an increase in fine fraction content and a decrease in rubber content. Different behavior was observed by Saberian et al. [29]. As shown in Figure 9b, RCA–RTW mixtures, show a change in volume like RCA, but they develop more dilation than pure RCA blends. The dilatant reaction is visible and the specimens are characterized by high susceptibility to volume change. This behavior indicates that the mixtures are in a highly compacted state. There is no doubt that the addition of rubber results in higher strain values, both volumetric and shearing strains. This is due to the deformable behavior of the rubber.

### 3.2. Shear Strength Parameters

Shear strength can be defined as the maximum internal resistance to the applied shearing force. The main parameters used in formulating the analysis of the shear strength of soil are the angle of internal friction, the so-called friction angle, and the cohesion. Many researchers have investigated the shear strength of tire waste mixed with natural soil using triaxial tests and/or direct shear tests [30,31].

The results of Humphrey and Sandford [32] from the direct shear tests on pure tire chips samples exhibit friction angles ranging from 20 to 35°, and cohesion from 3 to 11.5 kPa. The triaxial tests from Wu [11] produced higher values of φ, greater than 40° The majority of the studies conducted found that the inclusion of rubber chips in sand increased shear strength. On the other hand, reducing the particle size of rubber waste from chips to granulated rubber adversely affected the shear strength capacity [33]. For rubber-cohesive soil mixtures, opinions are divided as to the positive effect of rubber addition on the strength characteristics. Some believe that the addition of rubber chips to clay increased the shear modulus and cohesion intercept of the matrix material [34]. While others suggest that the shear strength remains the same or reduces with increasing rubber content, thereby implying that the bond between clayey soils and waste rubber is weak [35].

In Table 7, the strength parameters of soil-rubber mixtures for the composites obtained in the present study are presented. The given values of friction angle are similar to those for compacted noncohesive soils, fine sands, and silty sands, namely between 30° and around 42°. Corresponding values for RCA mixed with two different sizes and three different percentages of crumb rubber were reported by Saberian et al. [29]. The highest value of the effective angle of internal friction, around 42°, characterizes the mixture M8, the lowest, however, equal to φ = 30.20° was the mixture M5. Generally, the large values of the φ angle are most likely due to the high value of friction between grains. All tested mixtures were in an air-dry state. With an increase in moisture content, we could expect a decrease in friction values as the soil grains begin to be covered by a layer of water. It can be noticed as well that M1–M4 mixes have on average a value of the angle of internal friction of 39°. In the case of RCA–RTW blends (M5–M8 mixes), the obtained average value of the φ angle is slightly lower at 34.6°, which is about 11% of the difference.

In terms of cohesion, for the first four compositions, a decrease of this parameter is observed from a value of about 63 kPa for the mixture M1 to a value around half as low, i.e., 35 kPa for the mixture M4. The noticeably higher results of c are obtained for the M1 mixture, which has the highest cohesion of all four pure RCA mixes. It is an “apparent cohesion”, which is not realistic for dry unbounded granular materials. Even higher values of apparent cohesion, by 10 kPa, for recycled concrete aggregate were obtained by Perera et al. [36]. The specimens M1–M4 are characterized by an average cohesion value of 47 kPa. By analyzing the obtained values of c for the next four mixtures, the larger values of cohesion are found, with an average equal to 78 kPa. The average difference in the strength parameter c between specimens M5–M8 and M1–M4 is about 40% in favor of the former.

As presented in Table 7, rubber additive causes an initial increase in the strength parameter c by approx. 60 kPa (see specimen M7 compared to M8), followed by a decrease of ca. 34% (M6 compared to M7) and 14% (M5 compared to M6). This confirmed the observation of Saberian et al. [29], although these researchers obtained significantly smaller cohesive values. It is interesting to note that for the highest rubber content, i.e., the 20% of specimen M5, an average cohesion value is obtained. This was expected to be rather higher, due to the addition of rubber waste of a larger size. According to the findings of Anbazhagan et al. [28], who stated that larger size rubber particles have a good interlocking capacity, resulting in an increase in the cohesion with an increase in rubber particle size.

Generally, the higher decline of c in the mixture (see M5–M8 blends), the bigger their grain size and percentage composition. This fact can be explained by the decreasing domination of electromagnetic force between fine particles, which results in the separation of soil particles due to the increasing content of rubber waste. The disturbance of this trend is the mixture M8 containing 5% of powder rubber waste.

In Table 7, basic statistics for the strength parameters are summarized. The following parameters are calculated: average value, standard deviation, and standard error. It is worth noticing that the standard error for the mean friction angle values for all of the presented data is low, not exceeding the value of 4°. This confirms the regularity of the conducted research. On the other hand, the standard error values for the obtained cohesion values are larger, but still do not exceed 20 kPa.

The changes observed for strength parameters, φ, and c, for RCA and RCA-RTW mixtures are compiled in Table 8. The analysis of the φ values shows that the addition of rubber waste to RCA practically does not affect this parameter. It was expected that the friction angle increases with an increase in rubber content, or at least up to some percentage. The angularity of rubber contributes to increasing friction angle by interlocking with sand particles. According to Attom [37], the increase in the angle of internal friction is due to failure in the shearing zone. In this particular zone, the rubber particles are distributed and oriented randomly at the shearing surface. As shearing starts, the rubber particles either slide or resist the shearing against cut-off, which results in an increased shearing force.

Larger differences in the values are observed in the case of cohesion, though there is no consistency in the changes here. The values of cohesion are both increasing and decreasing. A noticeable decrease in apparent cohesion with the addition of recycled rubber can be explained by the fact that although rubber provided better shear strength for the RCA, due to its higher tensile strength, the increasing of the rubber content would lead to decreasing the shear parameters. A higher increase in apparent cohesion is achieved for the M7 mixture. This is a composition that contains 10% of rubber inclusion and 20% of fine fractions. This mix has therefore the highest shear strength.

In Table 8, the average densities (ρ_avg_) values are tabulated. The obtained values of ρ_avg_ prove that the addition of rubber waste reduces the density of RCA by approx. 2%, 6%, and 9% for specimens M7, M6, and M5, respectively. Smaller differences in density, ranging from 1% to about 4%, are observed for mixes without rubber with increasing fine fraction content. Changes in the fine fraction (mixtures M1–M4) result in half the changes in RCA density.

In general, the fine content significantly affects the engineering properties of noncohesive soils, if they consist of fine particles. In a recent study, Phan et al. [38] showed that as the fine content of sand–fine particle mixtures increased, all parameters of deviator stress, volumetric strain, shear stress, internal friction angle, and cohesion increased. Other researchers, however, have reported that as silt content increased, the steady-state strength initially decreased and subsequently increased in shear strength with further increases in the silt content to values greater than 30%.

**Figure 10:**
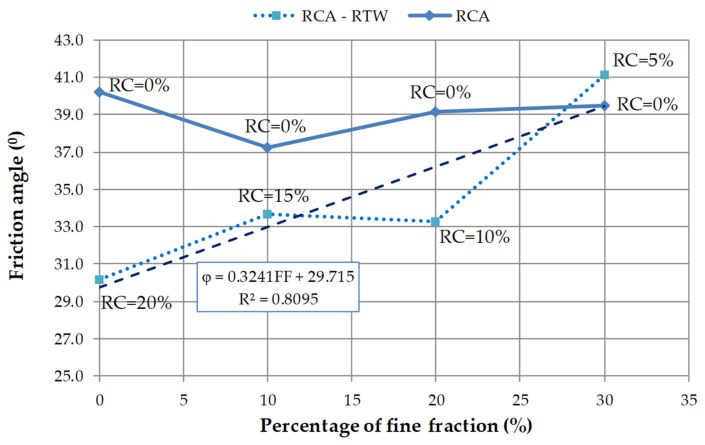
The relationship between friction angle and fine fraction of RCA and RCA–RTW mixtures. Note: RC—rubber content.

Figure 10 provides the relationship between friction angle and the fine content of all studied compositions. It is noticed that for mixtures of pure recycled concrete aggregate (M1–M4) there is a comparable situation to the one observed in the studies of other scientists. The initial decrease in the friction angle values is stopped for the M2 blend with 10% of the fine fraction of RCA. While for M4 mixture with 30% of fine content and increase of 6% in the strength parameter φ is observed, relative to the mixture M2. In the case of RCA–RTW mixtures, a strong linear relationship (R^2^ = 0.81) between friction angle and percentage of the fine fraction can be found. An increase in the fine fraction content by 10%, together with a decrease in the rubber additive by 5%, results in an increase in the value of the angle of internal friction by about 10°.

**Figure 11:**
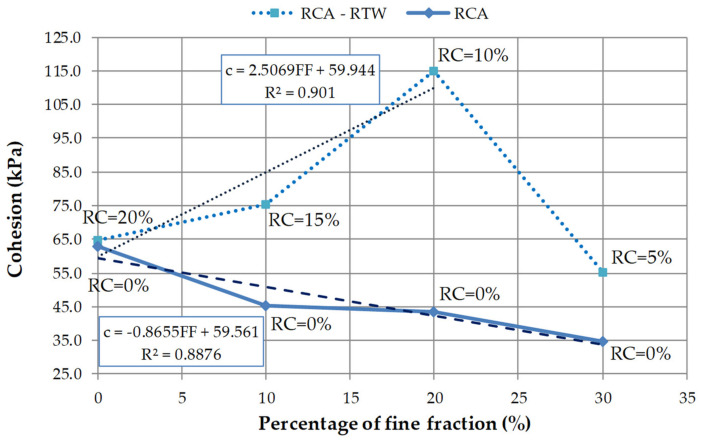
The relationship between cohesion and fine fraction of RCA and RCA–RTW mixtures. Note: RC—rubber content.

In Figure 11, the relationship between cohesion and the fines content of all studied compositions is shown. For RCA–RTW mixtures, a linear increase in the cohesion value with increasing fine fraction content, up to a value of FF = 20%, is observed. Mixture M7, with 20% of FF and 10% of RC has the highest cohesion, approx. 115 kPa. Mixture M8 is characterized, on the other hand, by the lowest cohesion value, ca. 55 kPa, although it has the highest percentage of the fine fraction—30%. In the case of testing M1–M4 mixtures, it can be concluded that the strength parameter c decreases with an increase in the fine fraction. For the proposed linear regression model R^2^ is equal to 0.89, which means that the variation of mix cohesion is explained by the variation of fine fraction content in almost 90%.

## 4. Conclusions and Recommendations for Further Research

In this study, a series of isotropic consolidation drained triaxial tests were performed to assess the selected mechanical properties of four recycled concrete aggregate mixtures with different fine fraction content and of four RCA mixed with different percentages of rubber waste. Despite the costs, it was decided to test such materials because they can effectively address the growing environmental concerns and at the same time provide solutions to geotechnical problems associated with, e.g., low soil shear strength and high dilatancy. The main conclusions of this research are:

1. All tested mixes have high mechanical properties. The values of the internal friction angles for the pure RCA compositions range from 37° to 40°, while for the RCA–RTW blends these values range from 30° to 41°. The mixes M1–M4 have on average an angle of internal friction of 39°. In the case of RCA–RTW blends (M5–M8 mixes), the obtained average value of the φ angle is slightly lower, at 34.6° (about 11% of the difference). The analysis of the φ values shows that the addition of rubber waste to RCA practically does not affect this parameter.
2. In the case of cohesion, the values ranging from 35 kPa to 63 kPa were obtained for specimens M1–M4, and from 55 kPa to 115 kPa for specimens M5–M8. The pure RCA specimens are characterized by an average cohesion value of 47 kPa. For the next four mixtures, enriched with a rubber additive, the larger values of cohesion are found, with an average equal to 78 kPa. The average difference in the strength parameter c between specimens M5–M8 and M1–M4 is about 40%, in favor of the former. The values of cohesion for RCA and RCA–RTW mixtures have various trends; they can fall or grow, initially increase and then decline, or vice versa.
3. With decreasing rubber content and increasing fine fraction content, the shear resistance of the mixtures increases by about 20% with decreasing shear strain at failure also by about 20%.
4. Based on the experimental results, it can be noticed that the inclusion of rubber in RCA can alter the shear strength, cohesion, friction angle, and volumetric strain. Generally, the improvement in RCA shear strength is observed. However, the largest improvement in shear strength occurs for the mixture M7, with 10% of recycled tire waste.
5. In this research, recycled concrete aggregate modified by rubber addition will have more strength at small strain values. The addition of rubber results in higher strain values, both volumetric and shearing strains. The magnitude of the shear strain at the peak deviator stress increases with the increasing proportion of rubber in the mixture. The highest values of the shear strain, regardless of the mean effective stress values, are observed for the M5 mixture, with 20% of rubber addition. As the rubber content of the specimen decreases by 5%, the strain decreases by an average of 8%.
6. The dilatancy characteristics of studied RCA blends are similar to the behavior of cohesionless material, having both negative and positive magnitudes of dilatancy. This is due to the deformable behavior of the rubber. Dilatant behavior is noticed because of the interlocking between the rubber and RCA particles. By increasing the mean effective stress, the tendency for dilation decreases. There is no significant reduction in RCA dilatancy due to the inclusion of rubber from recycled tires waste.
7. The addition of rubber waste reduces the density of RCA, by an average of 6%. Changes in the fine fraction (mixtures M1–M4) result in half the changes in RCA density.
8. The presented results are found to be significantly affected by the content of the fine fraction of RCA. An increase in the fine fraction content by 10%, together with a decrease in the rubber additive by 5%, results in an increase in the value of the angle of internal friction of about 10°. For RCA–RTW mixtures, a linear increase (around approx. 24%) in the cohesion value with increasing fine fraction content, up to a value of FF = 20%, is observed. For RCA mixtures the strength parameter c decreases with an increase in the fine fraction by 9 kPa on average.

Based on the results of all tests performed, including physical, geometric, chemical, and mechanical properties of the created composites, it can be stated that the samples would meet local road authority requirements for the subbase applications.

It is planned to perform further tests on RCA–RTW mixtures without FF content. On the other hand, it is worth checking the influence of other rubber dimensions (coarse rubber) on the strength characteristics of the tested mixtures. Moreover, the obtained research results can be developed as well by studying the change in the shear strength characteristics of the proposed blends influenced by such environmental impacts as rain, temperature drops, heaving, etc. This is because the road base is always exposed to these environmental effects.

## Figures and Tables

**Table 1:** Classification of materials obtained from waste tires according to EN-14243 [8].

Type of Shredded Rubber Waste	Particle Size in (mm)
Tire cuts (halves and smaller pieces)	>300
Shreds	50–300
Chips	10–50
Granulate	1–10
Powder	<1
Fine powder	<0.5
Scrap (a byproduct of tire retreading)	0–40

**Table 2:** Comparison of characteristics of recycled rubber materials with mineral aggregate [9].

Properties	Rubber Waste (Rubber Aggregate)	Rubber Packages	Mineral Aggregate
Bulk density (kN/m^3^)	5–7	7	18–20
Friction angle (°)	19–38	35–37	35–40
Cohesion (kPa)	0–11.5	0	0
Deformation modulus (MPa)	0.8–1.3	0.8–1.0	40–100
Water permeability (cm/s)	2–10	2–4	10–100
Thermal insulation (W/(m·K))	0.07	0.07	0.4–0.7
Vibration isolation	high	high	low

**Table 3:** Specifications of RCA mixtures.

Description	Mixtures No			
	1	2	3	4
Effective size, d_10_ (mm)	0.086	0.063	0.033	0.025
d_30_ (mm)	0.14	0.12	0.085	0.064
Mean size, d_50_ (mm)	0.20	0.16	0.14	0.12
d_60_ (mm)	0.25	0.18	0.17	0.14
Coefficient of uniformity, C_u_	2.91	2.86	5.15	5.60
Coefficient of curvature, C_c_	0.91	1.27	1.29	1.17
Minimum dry density, ρ_d min_ (g/cm^3^)	1.24	1.22	1.19	1.17
Maximum dry density, ρ_d max_ (g/cm^3^)	1.60	1.61	1.62	1.64

**Table 4:** Summary of test results for selected properties of concrete aggregate.

Description	Unit	Value
Sand index, SE	-	82
Shredding resistance, LA	%	39, LA_40_
Abrasion resistance, M_DE_	%	28, M_DE_35
Frost resistance, F	%	9.64, F10
CBR value	%	60
Flatness index, FI	%	42, FI_50_
Shape index, SI	%	23, SI_40_
Content of grains with crushed or broken surface	%	78
Grain density	Mg/m^3^	2.51
Water absorption	%	6.55
Bulk density loose	Mg/m^3^	1.41
Methylene blue	g/kg aggregate	1.66

**Table 5:** Chemical properties of RCA mixtures.

Spectrum	O	Na	Mg	Al	Si	P	S	K	Ca	Ti	Fe
Mass percent (%)											
Mean value	38.22	0.31	1.09	3.43	15.28	0.02	0.69	1.27	37.03	0.19	2.48
Standard deviation	1.62	0.13	0.20	0.34	3.38	0.02	0.15	0.16	4.41	0.03	0.29
Mean standard deviation	0.57	0.05	0.07	0.12	1.20	0.01	0.05	0.06	1.56	0.01	0.10

**Table 6:** Leachate concentration from RCA mixtures.

Element	Co (mg/L)	Ni (mg/L)	Cu (mg/L)	Zn (mg/L)	Cd (mg/L)	Pb (mg/L)	Cr (mg/L)	Sulphates (mg/L)	Chlorides (mg/L)	Specific Conductivity (μS/cm)	pH
Value											
M1_RCA_0FF	0.018	<0.015	0.061	0.546	0.036	0.035	<0.03	43	5.0	144.2	9.55
M2_RCA_10FF	0.018	<0.015	0.067	0.541	<0.008	0.015	<0.03	55	15.0	456	9.70
M3_RCA_20FF	0.013	<0.015	0.045	0.520	<0.008	<0.015	<0.03	155	7.0	444	10.2
M4_RCA_30FF	0.015	<0.015	0.075	0.852	<0.008	<0.015	<0.03	250	8.5	666	9.79
Acceptance criteria *	1	0.5	0.5	2	0.05	0.5	0.5	500	1000		

* Official Gazette of the Republic of Poland, Regulation of the Minister of the Environment of 18 November 2014 on the conditions to be met for the introduction of sewage into waters and to land and on substances particularly harmful to the aquatic environment.

**Table 7:** Results of strength parameters and basic statistics for studied materials.

Mixture No	Material	Rubber Content (%)	Friction Angle (°)			Cohesion (kPa)		
			Average	Std. Dev.	Std. Error	Average	Std. Dev.	Std. Error
1	RCA_0FF	0	40.19	4.94	2.0	63.01	46.62	20.8
2	RCA_10FF	0	37.21	8.23	3.7	45.17	24.80	11.1
3	RCA_20FF	0	39.17	0.94	0.5	43.37	9.27	5.4
4	RCA_30FF	0	39.46	0.65	0.4	34.76	6.36	3.7
5	RCA_20R_0FF	10G ^1^, 10P ^2^	30.20	1.28	0.7	64.74	10.20	5.9
6	RCA_15R_10FF	15P ^2^	33.69	3.09	1.8	75.42	27.93	16.1
7	RCA_10R_20FF	10P ^2^	33.26	2.84	1.6	114.88	27.79	16.0
8	RCA_5R_30FF	5P ^2^	41.15	1.95	1.0	55.23	33.12	16.6

^1^ Granulate rubber waste; ^2^ powder rubber waste.

**Table 8:** Variations of strength parameters and results of density for studied materials.

Mixture No	Material	Rubber Content (%)	The Ratio of Improvement/Reduction in		Average Density (g/cm^3^)
			Friction Angle (-)	Cohesion (-)	
1	RCA_0FF	0	-	-	1.65
2	RCA_10FF	0	0.93	0.72	1.63
3	RCA_20FF	0	1.05	0.96	1.55
4	RCA_30FF	0	1.01	0.80	1.52
5	RCA_20R_0FF	10G, 10P	-	-	1.23
6	RCA_15R_10FF	15P	1.12	1.16	1.35
7	RCA_10R_20FF	10P	0.99	1.52	1.43
8	RCA_5R_30FF	5P	1.24	0.48	1.47

## Data Availability

Data available on request due to their size properties. The data presented in this study are available on request from the corresponding author.
